# Combination of cell signaling molecules can facilitate *MYOD1*-mediated myogenic transdifferentiation of pig fibroblasts

**DOI:** 10.1186/s40104-021-00583-1

**Published:** 2021-05-13

**Authors:** Jinsol Jeong, Kwang-Hwan Choi, Seung-Hun Kim, Dong-Kyung Lee, Jong-Nam Oh, Mingyun Lee, Gyung Cheol Choe, Chang-Kyu Lee

**Affiliations:** 1 Department of Agricultural Biotechnology, Animal Biotechnology Major, and Research Institute of Agriculture and Life Science, Seoul National University, Seoul, 08826 South Korea; 2 Present address: Research and Development Center, Space F corporation, Hwasung-si, Gyeonggi-do 18471 South Korea; 3 Institute of Green Bio Science and Technology, Seoul National University, Pyeong Chang, Kangwon-do 25354 South Korea

**Keywords:** MYOD1, Pig, Sequence analysis, Skeletal muscle, Transdifferentiation

## Abstract

**Background:**

Myogenic transdifferentiation can be accomplished through ectopic *MYOD1* expression, which is facilitated by various signaling pathways associated with myogenesis. In this study, we attempted to transdifferentiate pig embryonic fibroblasts (PEFs) myogenically into skeletal muscle through overexpression of the pig *MYOD1* gene and modulation of the FGF, TGF-β, WNT, and cAMP signaling pathways.

**Results:**

The *MYOD1* overexpression vector was constructed based on comparative sequence analysis, demonstrating that pig MYOD1 has evolutionarily conserved domains across various species. Although forced *MYOD1* expression through these vectors triggered the expression of endogenous muscle markers, transdifferentiated muscle cells from fibroblasts were not observed. Therefore, various signaling molecules, including FGF2, SB431542, CHIR99021, and forskolin, along with *MYOD1* overexpression were applied to enhance the myogenic reprogramming. The modified conditions led to the derivation of myotubes and activation of muscle markers in PEFs, as determined by qPCR and immunostaining. Notably, a sarcomere-like structure was observed, indicating that terminally differentiated skeletal muscle could be obtained from transdifferentiated cells.

**Conclusions:**

In summary, we established a protocol for reprogramming *MYOD1*-overexpressing PEFs into the mature skeletal muscle using signaling molecules. Our myogenic reprogramming can be used as a cell source for muscle disease models in regenerative medicine and the production of cultured meat in cellular agriculture.

## Background

To date, numerous studies have defined various strategies for the differentiation of PSCs (pluripotent stem cells) into specific cell types of ecto-, meso-, and endodermal lineages, which have advantages as infinite cell sources [1–3]. Whereas these directed differentiation approaches recapitulate *in vivo* developmental processes through modulation of signaling pathways, they reportedly have defects in terms of time consumption and low efficiency because the small molecules used are also involved in differentiation into other cell types [4, 5]. For example, the derivation of striated myofibers, a form of mature skeletal muscle, from mouse ESCs (embryonic stem cells) and human iPSCs (induced pluripotent stem cells) reportedly takes 3–4 weeks, albeit with the use of an advanced protocol with a short experimental period [6, 7]. Therefore, direct reprogramming using the activation of the transcriptional program has been applied to facilitate the differentiation of PSCs into the desired cell type [5]. However, problems derived from the use of PSCs still remain, including the potential risk of teratoma formation [8] and the requirement of a time-consuming and fine-tuning process for the derivation and maintenance of the pluripotent state. To overcome the above problems, a transdifferentiation approach is required, in which fully differentiated somatic cells, instead of PSCs, are induced to differentiate directly into target cell types via ectopic expression of transcription factors. Transdifferentiation toward the muscle lineage has been widely studied for a long time since the identification of a master transcription factor in myogenesis, Myod1 [9].

Forced *Myod1* expression converts cell fates into muscle, which is responsible for Myod1 functioning as a transcriptional and epigenetic regulator, leading to the activation of muscle-specific genes in a feed-forward manner [10, 11]. Small molecules can be supplemented in culture conditions for *Myod1/MYOD1*-overexpressing somatic cells to improve the efficacy of transdifferentiation. The surface of myocytes and the basement membrane of myotubes secrete the FGF2 (fibroblast growth factor 2), which play roles in the activation of muscle stem cells and the expansion of myoblasts, inhibiting terminal differentiation of muscle [12, 13]. Because the TGF-β signaling negatively functions in myogenesis by decreasing Myog activity, inhibitor of TGF type I-β receptors, such as SB431542, reportedly enhances the elongation of myotubes through myoblast fusion [13]. In fact, the expression of follistatin, which inhibits the TGF-β signaling, is detected in the paraxial mesoderm *in vivo* [14]. During *in vivo* myogenesis, the WNT activator secreted from the notochord, neural tube, and surrounding tissue is involved in a series of specifications along with the presomitic mesoderm, somite, dermomyotome, and myotome, as determined by *in vitro* directed differentiation in human and mouse PSCs [3, 6, 7]. It demonstrates that the WNT signaling induces the commitment of myogenic precursors and has been substantiated by previous research showing that the WNT activator CHIR99021 with SB431542 and FGF2 induces myogenic specification from human iPSCs [14]. In a previous report, the adenylyl cyclase activator forskolin was identified as a myoblast proliferation-promoting factor through CREB-mediated WNT, leading to upregulation of the *Pax3*, *Myf5*, and *Myod1* genes [15]. The combination of FGF2, a WNT activator, and forskolin stimulated skeletal muscle differentiation in human iPSCs and especially forskolin improved satellite cell expansion in mice [16].

In pigs, myogenic reprogramming could be used as a cell source for the muscle disease modeling and the production of cultured meat. Although, in pig iPSCs, skeletal myotubes were reportedly observed through the activation of WNT signaling and ectopically expressed *MYOD1* by overexpression vector and 5-azacytidine (5-aza) [17], the aforementioned limitations derived from iPSCs remain. Moreover, it has been demonstrated that muscle stem cells lose the potential for proliferation and differentiation in long-term culture *in vitro* [18]. As an alternative, fibroblasts can be obtained in high yield because of their large proportion in the body and are known to be the most effective cell type to accept the expression of *Myod1* because they are derived from the mesoderm, which is identical in origin to the muscle [19]. Therefore, in this study, we attempted to transdifferentiate pig fibroblasts into skeletal muscle through overexpression of the pig *MYOD1* gene and modulation of the FGF, TGF-β, WNT, and cAMP signaling pathways.

## Methods

### Amino acid sequence analysis

The MYOD1 amino acid sequences of pig (NP_001002824.1), mouse (NP_034996.2), human (NP_002469.2), horse (NP_001304182.1), cow (NP_001035568.2), and sheep (NP_001009390.1) were obtained from the NCBI database (https://www.ncbi.nlm.nih.gov/). Comparative analysis of the amino acid sequences was performed using the multiple sequence alignment program MUSCLE (MUltipleby https://www.ebi.ac.uk/Tools/msa/muscle/), and the similarity of proteins from pigs and the other species was analyzed using Protein BLAST (https://blast.ncbi.nlm.nih.gov/).

### Construction of inducible p*MYOD1*-overexpression vector

Total RNA was extracted from satellite cells from the biceps femoris of 3-day-old LYD pigs based on our previous study [20], and cDNA was subsequently synthesized. Nested PCR was performed using an outer primer containing 20-bp sequences upstream and downstream of the *MYOD1* gene and an inner primer containing the EcoR1 sequence. The following primer set was used: Outer-*MYOD1*-F, 5′-ATAGAGCAGGGTGGTGGACA-3′, Outer-*MYOD1*-R, 5′-CTCAAACTTCTGGGCGCGAG-3′, Inner-*MYOD1*-F, 5′-GAATTCTGGGATATGGAGCTGCTGTCGC-3′, Inner-*MYOD1*-R, 5′-GAATTCTCAGAGCACCTGGTAGATAGGGGTTGG-3′. The PCR product was purified using electrophoresis and inserted into a T easy vector (Promega, Madison, WI, USA). DNA was extracted from the selected vectors through TA cloning and was sequenced using the following M-13 primer: F, 5′-GTAAAACGACGGCCAG-3′, R, 5′-CAGGAAACAGCTATGAC-3′. Subsequently, a doxycycline (DOX)-inducible FUW-tetO-p*MYOD1* vector was constructed using treatment with the restriction enzyme EcoR1 and the FUW-tetO-MCS vector (plasmid #84008; Addgene, Watertown, MA, USA).

### Lentiviral vector production

Lentiviral vectors were produced as previously described [21]. Briefly, HEK 293 LTV cells (Cell Biolabs, San Diego, CA, USA) were used as the packaging cell line, and five plasmids were used for the production of lentiviral vectors: FUW-tetO-p*MYOD1* and FUW-M2rtTA (the transfer plasmid); pLP1 and pLP2 (the packaging plasmids; Invitrogen, Waltham, MA, USA); and pLP/VSVG (the envelope plasmid; Invitrogen). These plasmids were transfected into HEK 293 LTV cells using the calcium phosphate precipitation method. Subsequently, the LTV culture supernatants were filtered and concentrated. The derived virus pellets were stored at − 76 °C until use.

### Myogenic transdifferentiation of pig fibroblasts

The pig embryonic fibroblasts (PEFs) isolated in previous study were used [22]. The cells were cultured in Dulbecco’s modified Eagle’s medium (DMEM; Welgene, Gyeongsan, Korea) supplemented with 10% fetal bovine serum (FBS; collected and processed in the United States; Genedepot, Katy, TX, USA), 1× GlutaMAX (Gibco, Gaithersburg, MD, USA), 0.1 mmol/L β-mercaptoethanol (Gibco), and 1× antibiotic/antimycotic (Gibco). The exogenous gene was transduced with a lentivirus carrying the FUW-tetO-p*MYOD1* or FUW-M2rtTA plasmid. The cells were incubated with lentivirus using 8 mg/mL polybrene (Sigma-Aldrich, St. Louis, MO, USA) for 48 h.

The virus-infected cells were cultured under various conditions, which consisted of basal medium and signaling molecules. The basal medium was composed of DMEM, 10% FBS, 10% knockout serum replacement (KSR; Gibco), 10% 1× GlutaMAX, 0.1 mmol/L β-mercaptoethanol, and 1× antibiotic/antimycotic. The tested signaling molecules were 20 ng/mL fibroblast growth factor 2 (FGF2; R&D Systems, Minneapolis, MN, USA), 4 μmol/L TGF-β receptor inhibitor SB431542 (Cayman chemical, Ann Arbor, MI, USA), 3 μmol/L GSK3B inhibitor CHIR99021 (Cayman chemical), and 10 μmol/L cAMP activator forskolin (Cayman chemical). 4 ng/mL DOX was added to activate the inducible vector. In the ‘differentiation step’, each medium was replaced with a serum-free basal medium containing 2% horse serum. The media were changed every day, and all cells were cultured under humidified conditions with 5% CO_2_ at 37 °C.

### Genomic DNA (gDNA) extraction and polymerase chain reaction (PCR)

Genomic DNA was extracted using the G-spin™ Total DNA Extraction Kit (iNtRON, Seongnam, Korea). Amplifications were performed using the transgene-specific primers listed in Table 1 and 2×PCR Master mix solution (iNtRON) containing 5 pmol of each primer set and 50 ng gDNA in a 10-μL reaction volume. PCRs were performed in a thermocycler under the following conditions: 94 °C for 5 min, followed by 35 cycles of denaturation at 95 °C for 30 s, annealing for 30 s (annealing temperatures depended on each primer set), and extension at 72 °C for 30 s, with a final extension at 72 °C for 7 min. The amplified PCR products were visualized using electrophoresis on a 1% agarose gel stained with ethidium bromide.

**Table 1:** Primer sets used for PCR and qPCR

Gene	Primer sequence (5'→3')		Annealing temperature, °C	Product size, bp
***FUW-TetO-pMYOD1 (ExoMYOD1)***	F	CCAGGTGCTCTGAGAATTCGATA	60	114
	R	CCACATAGCGTAAAAGGAGCA		
***EndoMYOD1***	F	AGGGACAGGATAGAGCAGGG	60	199
	R	TCAAATCTACGTCGCGGAGC		
***PAX7***	F	GTGCCCTCAGTGAGTTCGAT	58	152
	R	TCCAGACGGTTCCCTTTGTC		
***MYF5***	F	AGTTCGGGGACGAGTTTGAG	60	232
	R	TCAAACGCCTGGTTGACCTT		
***MYOG***	F	GAGCTGTATGAGACATCCCCC	60	75
	R	GTGGACGGGCAGGTAGTTTT		
***MHC***	F	ACAGTGAAGACGGAAGCAGG	60	153
	R	TGCGTAACGCTCTTTGAGGT		
***pACTB***	F	CCGGGACCTGACCGACTACC	60	126
	R	TCGAAGTCCAGGGCGACGTA		
***GAPDH***	F	TGCTCCTCCCCGTTCGAC	60	100
	R	ATGCGGCCAAATCCGTTC		

### Immunocytochemistry (ICC) analysis

Before staining, all cell samples were preincubated for 5 min at 4 °C and fixed with 4% paraformaldehyde for 15 min. After washing twice with Dulbecco’s phosphate-buffered saline (DPBS; Welgene), the samples were treated for 1 h with 10% goat serum in DPBS to prevent nonspecific binding. Serum-treated cells were incubated overnight at 4 °C with primary antibodies. The primary antibodies used were as follows: rabbit anti-MYOD1 (1:100, Thermo Fisher, Waltham, MA, USA; PA5-23078) and mouse anti-MHC (1:50, Sigma-Aldrich; 05-716). When antibodies against MYOD1 were applied, fixed cells were treated for 15 min with 0.2% Triton-X100 (Sigma-Aldrich) before serum blocking. After incubation with the primary antibody, the cells were treated for 2 h at room temperature with an Alexa Fluor-conjugated secondary antibody. Nuclei were stained with Hoechst 33342 (Molecular Probes, Eugene, OR, USA). Images of stained cells were captured using a TE2000-U inverted microscope (Nikon, Tokyo, Japan).

### Quantitative real-time polymerase chain reaction (qPCR)

Total RNA was extracted from the cells using TRIzol^Ⓡ^ Reagent (Invitrogen) according to the manufacturer’s instructions. cDNA was synthesized using the High Capacity RNA-to-cDNA Kit (Applied Biosystems, Waltham, MA, USA), producing a final volume of 20 μL. The derived cDNA samples were amplified with PowerSYBR^Ⓡ^ Green PCR Master Mix (Applied Biosystems) containing 0.5 pmol of each primer set listed in Table 1 in a 10-μL reaction volume. Amplification and detection were conducted using the ABI 7300 Real-Time PCR system (Applied Biosystems) under the following conditions: one cycle of 50 °C for 2 min and 95 °C for 10 min, followed by 40 or 45 cycles of denaturation at 95 °C for 15 s and annealing/extension for 1 min (annealing/extension temperatures depended on each primer set). The relative expression level was calculated by normalizing the threshold cycle (Ct) values of each gene to that of *GAPDH* via the Δ–Ct method [23].

### Statistical analysis

The data from the qPCR analyses are presented as the mean ± standard error of the mean (SEM) and were analyzed using Prism 6 software (GraphPad Software; San Diego, CA, USA). The significance of differences was determined by one-way analyses of variance followed by Fisher’s least significant difference test. Differences were considered significant at *P* < 0.05.

## Results

### Pig *MYOD1* overexpression vector construction

**Fig. 1:**
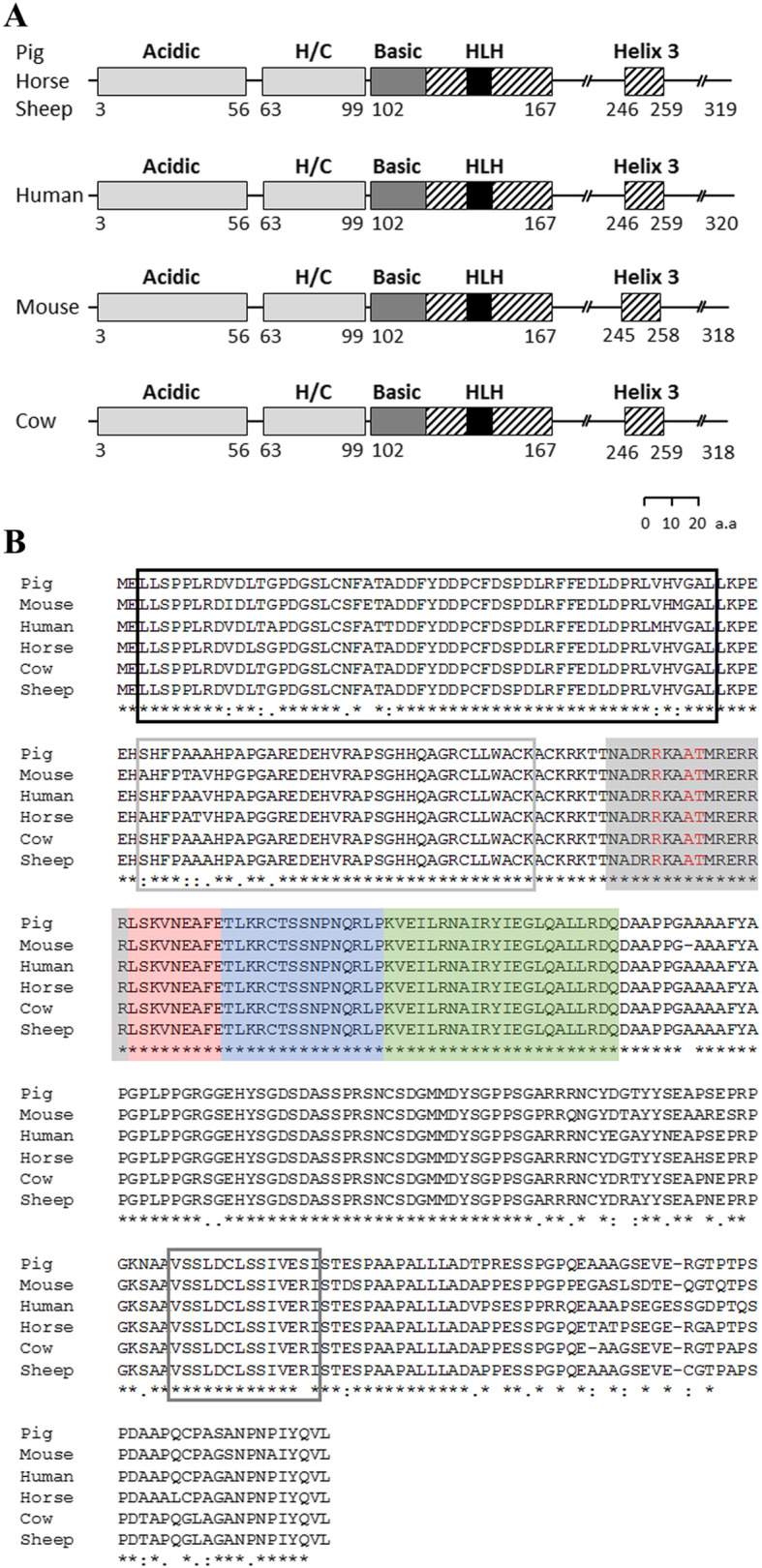
MYOD1 amino acid sequence analysis. The MYOD1 amino acid sequences of the pig (NP_001002824.1), mouse (NP_034996.2), human (NP_002469.2), horse (NP_001304182.1), cow (NP_001035568.2), and sheep (NP_001009390.1) were acquired from the NCBI database. **a** Schematic representation of the MYOD1 protein. The whole protein (black line) and its acidic and H/C domains (light gray boxes), basic domain (dark gray box), helix motifs (slashed boxes), and loop motif (black box) are indicated. The diagram is scaled. **b** Alignment and comparison of the MYOD1 amino acid sequence via the multiple sequence alignment program MUSCLE. The acidic domain (black line), H/C domain (light gray line), basic domain (gray box), helix1 (red box) -loop (blue box) -helix2 (green box), and helix3 motif (dark gray line) are indicated in the amino acid alignment. Arg^111^ and Ala^114^-Thr^115^ are marked in red. The consensus sequence of amino acids among the species is described with an asterisk at the bottom line

Myod1/MYOD1 is reportedly identified as a master transcription factor in myogenesis, thereby inducing myogenic transdifferentiation in non-muscle cells [9, 24–27]. Thus, a comparative MYOD1 sequence analysis was performed among various species to assess whether pig MYOD1 contains functionally conserved sequences for myogenesis, as in the other species, before vector construction for ectopic pig *MYOD1* (p*MYOD1*) expression. The pig MYOD1 whole protein has a similar size to MYOD1 protein in other species (Fig. 1A). To all the analyzed species, including pig, the MYOD1 protein contains acidic domains, histidine and cysteine-rich (H/C) domains, basic-helix1-loop-helix2 (bHLH) domains, and helix3 domains [28, 29]. Subsequently, the MYOD1 sequences from those species were aligned, and the similarity score was assessed at the amino acid level (Fig. 1b and Table 2). In the bHLH domain, Ala^114^-Thr^115^ and Arg^111^ reportedly endow the MYOD1 protein with myogenic activity [30, 31]. In fact, these residues (marked in red; Fig. 1b) were confirmed to be conserved in the basic region of all the analyzed species. It is noteworthy that the bHLH domain showed a 100.00% similarity between pig and all other species. Altogether, porcine MYOD1, especially the bHLH domain, was identified as an evolutionarily conserved protein, which seems that its role is also conserved in myogenesis across species.

**Table 2:** Similarity analysis of the pig MYOD1 amino acid sequence compared to other species

	Similarity between pig and the other species, %				
	Mouse	Human	Horse	Cow	Sheep
**MYOD1 protein**	89.03	93.12	94.36	95.92	95.61
**Acidic**	92.59	92.59	98.15	100.00	100.00
**H/C**	89.19	97.30	89.19	100.00	100.00
**bHLH**	100.00	100.00	100.00	100.00	100.00
**Helix3**	92.86	92.86	92.86	92.86	92.86

Similarity comparison between pig and the other species was confirmed on the score via the Protein BLAST program

**Fig. 2:**
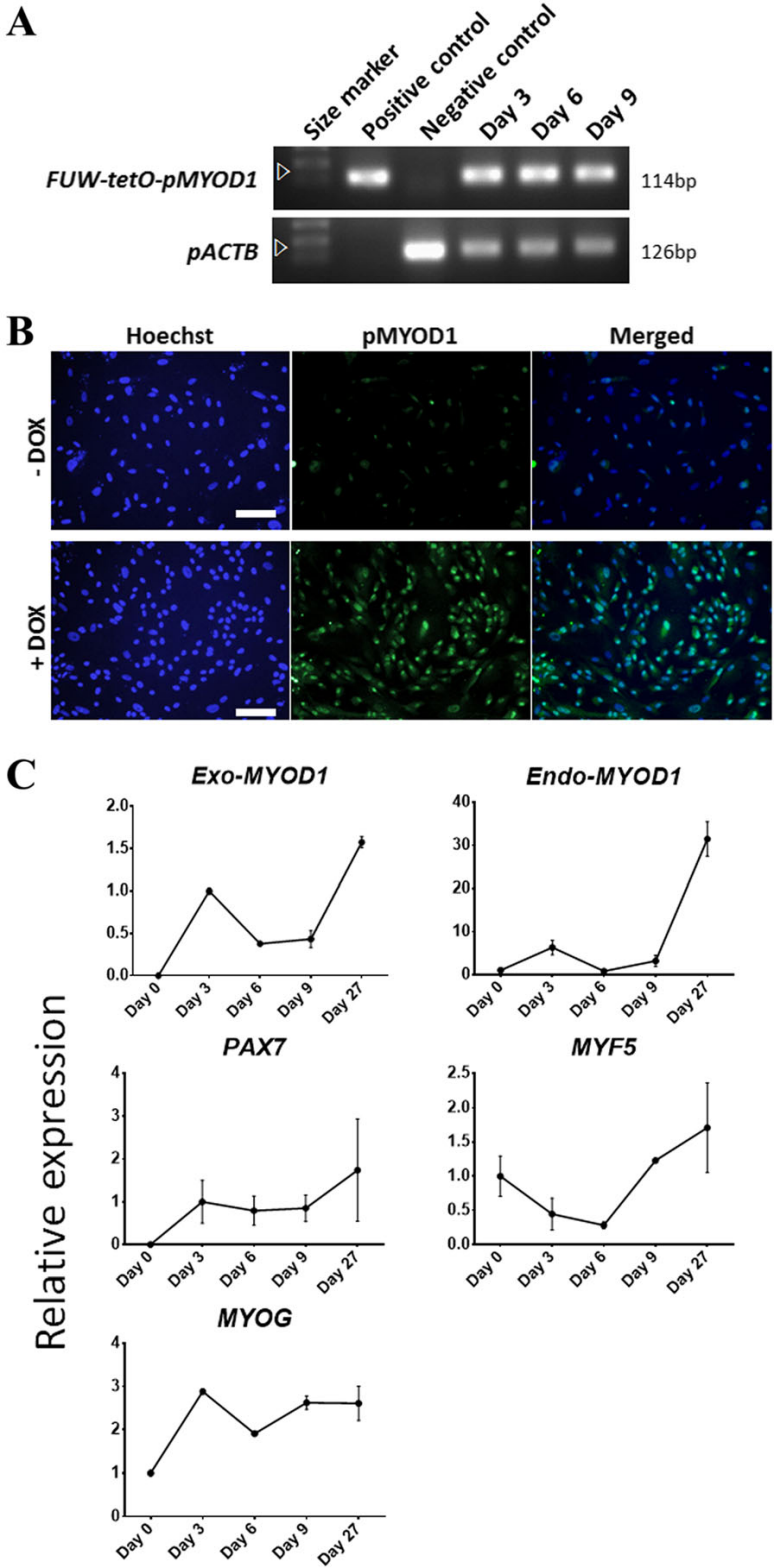
Vector construction and validation using transfection into PEFs. Construction of an inducible vector (FUW-tetO-p*MYOD1*) carrying the *MYOD1* gene isolated from satellite cells in 3-day-old LYD biceps femoris. Cells were cultured in a serum-free basal medium containing 2% horse serum and 4 ng/mL doxycycline (DOX). **a** PCR results using genomic DNA (gDNA) from PEFs transfected with FUW-tetO-p*MYOD1* to confirm the integration of the exogenous p*MYOD1* gene. Cells were sampled on days 3, 6, and 9 of DOX treatment. The FUW-tetO-p*MYOD1* plasmid was a positive control, and PEFs without transfection were the negative control. The arrows indicate the FUW-tetO-p*MYOD1* plasmid vector and loading control pACTB (porcine beta-actin; internal protein). **b** Immunofluorescence images for p*MYOD1* in PEFs transfected with FUW-tetO-p*MYOD1*. Scale bar = 100 μm. **c** qPCR results of PEFs transfected with the FUW-tetO-p*MYOD1* vector to confirm the expression patterns of muscle-associated genes (*Exo-MYOD1, Endo-MYOD1, PAX7, MYF5,* and *MYOG*). Cells on day 0 were used as a negative control. Relative gene expression is represented as a trend line, describing 1 as the value of day 0 in *Endo-MYOD1, MYF5*, and *MYOG* and the value of day 3 in *Exo-MYOD1* and *PAX7*

Based on the above analyses, doxycycline (DOX)-inducible p*MYOD1* overexpression vectors were generated including the *MYOD1* gene isolated from satellite cells in 3-day-old LYD biceps femoris. To verify the function of these constructed vectors, they were introduced into pig embryonic fibroblasts (PEFs) through lentiviral infection for stable transgene expression [4, 27]. First, the integration of the exogenous p*MYOD1* gene was confirmed by PCR targeting the FUW-tetO-p*MYOD1* sequence in gDNA of PEFs infected with a lentivirus carrying the vectors (p*MYOD1*-PEFs) (Fig. 2a). At day 9, the gene of interest had been inserted stably into the genome of PEFs. Then, MYOD1 expression was confirmed at the protein level using immunostaining in p*MYOD1*-PEFs (Fig. 2b). The vectors were activated by the addition of DOX, leading to ectopic *MYOD1* expression. Finally, the expression pattern of muscle-associated genes (*Exo-MYOD1, Endo-MYOD1, PAX7, MYF5*, and *MYOG*) was analyzed in p*MYOD1*-PEFs using qPCR (Fig. 2c). These genes have been characterized as myogenic lineage-specific markers, such as skeletal muscle progenitor/myoblasts (*PAX7, MYF5*, and *MYOD1*) and myocytes (*MYOD1* and *MYOG*) [3]. *Exo-MYOD1* overexpression by vector activation increased the expression of endogenous muscle-associated genes. During extended cell culture, the expression of these genes was stably maintained. In conclusion, we constructed a DOX-inducible p*MYOD1* overexpression vector that triggered the expression of muscle markers in long-term culture, indicating its stable function.

### Myogenic transdifferentiation of PEFs through overexpression of *MYOD1* and cell signaling modulation

The expression of endogenous muscle markers, including *Endo-MYOD1*, was enhanced via ectopic *MYOD1* expression, as shown in Fig. 2c. However, because of the mild changes in each gene, transdifferentiated muscle cells from fibroblasts were not observed. It has been shown that complete transdifferentiation is achieved by genetic modulation along with suitable culture conditions for specific cell types [32], suggesting that optimization of culture conditions is required. According to previous studies, a 2-step transdifferentiation protocol was employed with some modification to derive myogenic cells through *MYOD1* overexpression [8, 17, 33]. In the ‘induction’ step, the myogenic program was activated with the stimulation of transcription factors associated with myogenesis, thereby leading to the commitment into a myogenic lineage. Briefly, p*MYOD1*-PEFs were treated with various signaling molecules, such as FGF2, SB431542, CHIR99021, and forskolin, which are involved in the regulation of myogenesis [8, 14]. Then, transdifferentiation was promoted through serum starvation in the ‘differentiation’ step.

**Fig. 3:**
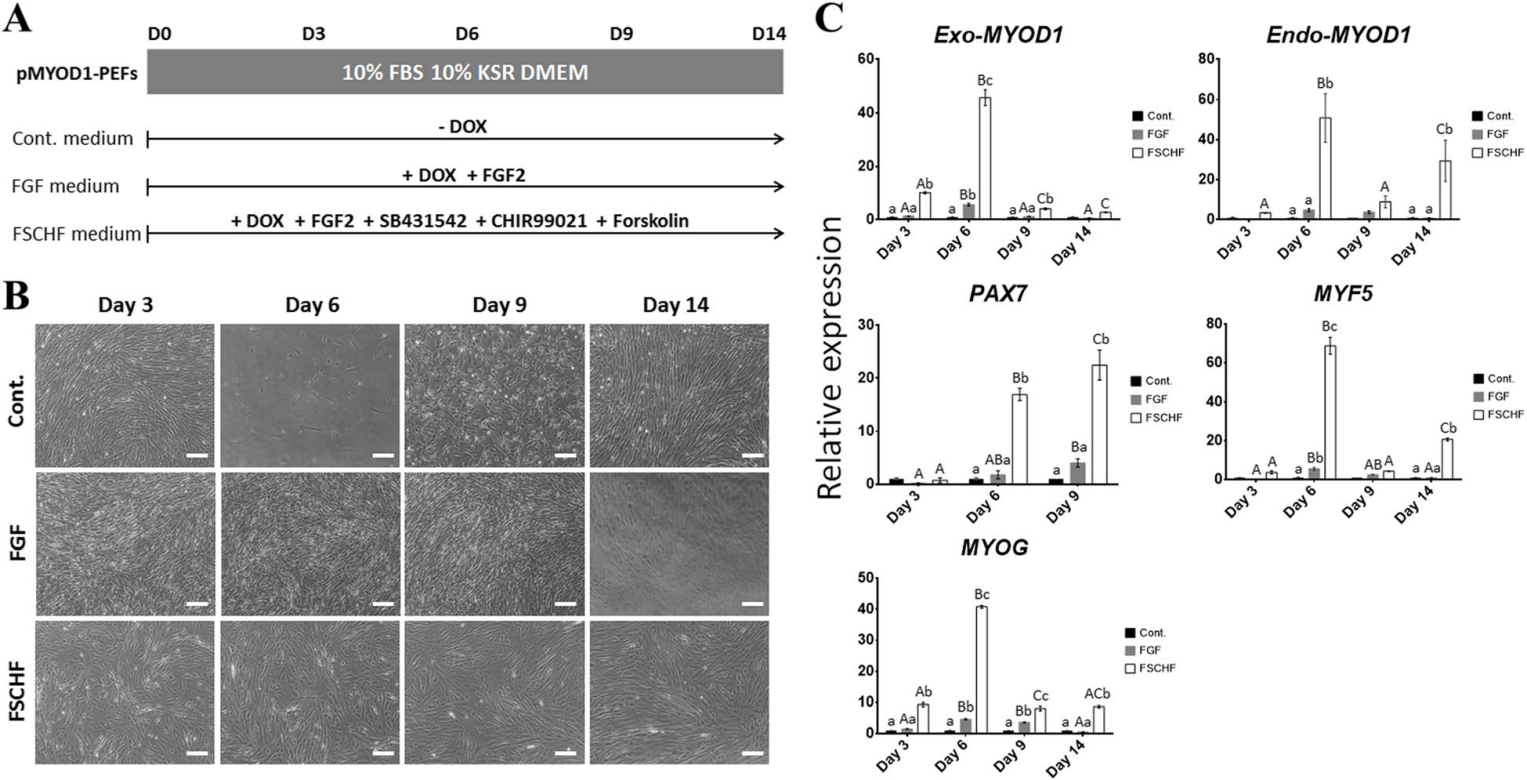
Induction of the myogenic program without entering the differentiation process. **a** Experimental scheme. Three groups (Cont. medium, FGF medium, and FSCHF medium) of p*MYOD1*-PEFs were induced toward the myogenic lineage according to the respective culture conditions for 14 days. Cont.: Control, FSCHF: FGF2, SB431542, CHIR99021, and forskolin. **b** Cell images showing the morphological changes under the respective culture conditions. Scale bar = 200 μm. **c** qPCR results to confirm the expression patterns of muscle-associated genes (*Exo-MYOD1, Endo-MYOD1, PAX7, MYF5,* and *MYOG*) in p*MYOD1*-PEFs. The amount of gene expression in the Cont. group is described as 1 compared to the other groups. Lowercase letters indicate comparisons among the 3 groups distinguished by the composition of signaling molecules (Cont., FGF, and FSCHF) for each culture period (days 3, 6, 9, or 14). Uppercase letters indicate comparisons among the culture periods (day 3 × day 6 × Dday 9 × day 14) for each group. Significant differences are represented by different letters, *n* = 3

To ensure efficient myogenic conversion, we investigated the transition point where exogenous *MYOD1* leads to the peak expression of endogenous skeletal muscle-specific genes. In addition, p*MYOD1*-PEFs were cultured in mitogen-rich media in which myogenic induction was sustained without entering the differentiation process (Fig. 3a). While the FSCHF group had a long cylindrical shape due to elongation of the cytoplasm, a typical fibroblastic and round shape was observed in the control and FGF groups, respectively (Fig. 3b). The alteration in the FSCHF group was maintained by the end of the culture period, resulting in a similar morphology to myoblasts, as previously reported [17, 34]. The myogenic genes were upregulated by forced *MYOD1* expression (Fig. 3c). Compared to other groups, the relative gene expression of the FSCHF group was higher across all the genes and culture periods. These results suggested that the *Exo-MYOD1* effect was enhanced by the combination of four signaling molecules rather than DOX-induced exogenous *MYOD1* per se or additional FGF2. While the gene expression of the FGF group was gradually changed, that of the FSCHF group was significantly increased on day 6 and then decreased. The aforementioned expression patterns were observed in all the markers except *PAX7,* which was upregulated up to day 9 in both groups. Taken together, p*MYOD1*-PEFs treated with a cocktail of FGF2, SB431542, CHIR99021, and forskolin for 6 days were used for further experiments.

**Fig. 4:**
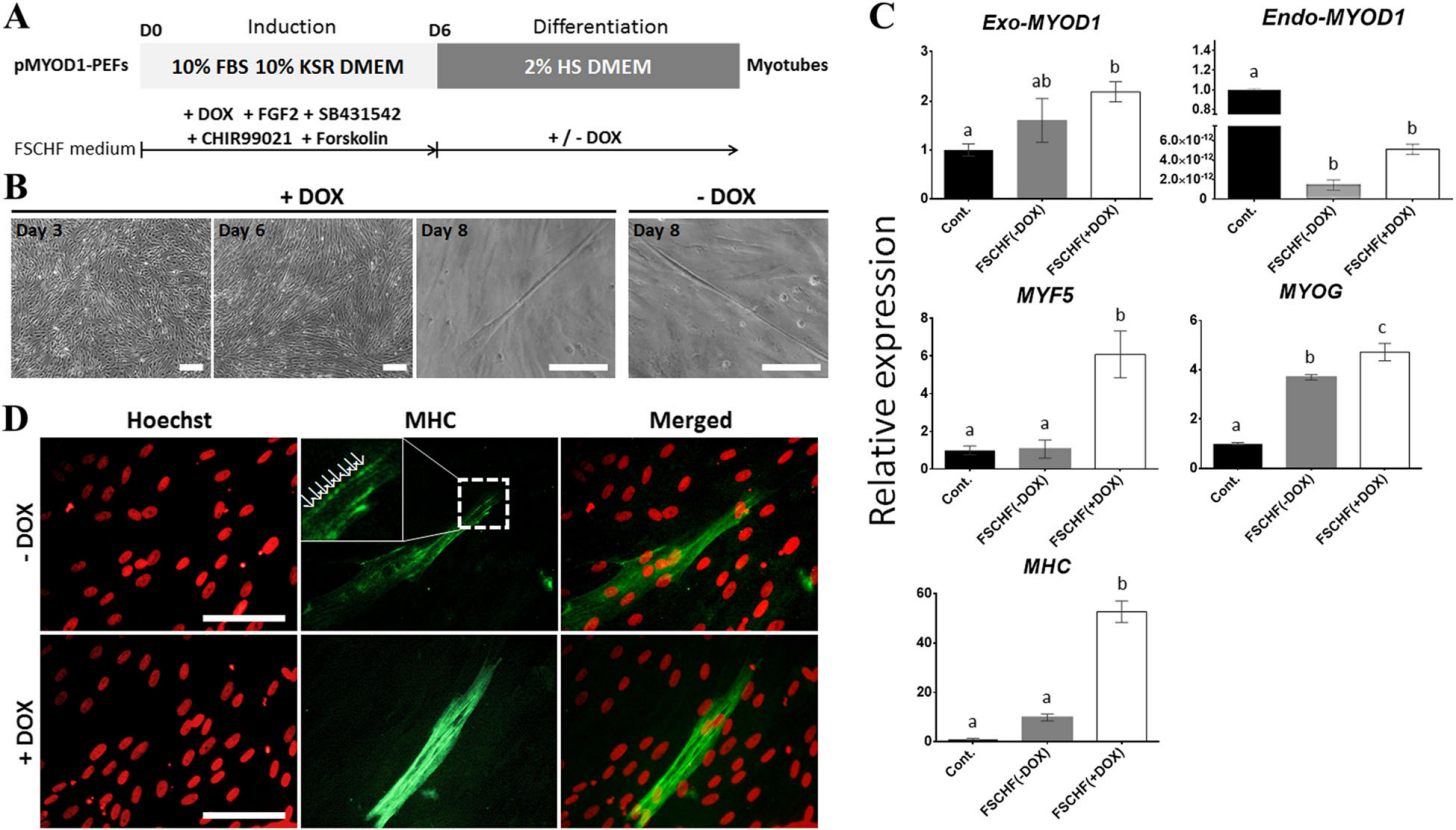
Transdifferentiation of p*MYOD1*-PEFs into myotubes using signaling molecules. **a** Experimental design for the transdifferentiation of p*MYOD1*-PEFs into myocytes. The p*MYOD1*-PEFs were induced toward the myogenic program in the ‘induction’ step, and the induced cells were promoted for myogenic reprogramming in the ‘differentiation’ step. In the differentiation stage, one group was treated with DOX, and the other was not. HS: horse serum, Cont.: Control, FSCHF: FGF2, SB431542, CHIR99021, and forskolin. **b** Cell images under culture conditions with or without DOX. Scale bar = 200 μm. **c** qPCR results to confirm the expression patterns of muscle-associated genes (*Exo-MYOD1, Endo-MYOD1, MYF5, MYOG,* and *MHC*) in p*MYOD1*-PEFs. The Cont. group contained p*MYOD1*-PEFs cultured in basal media. The amount of gene expression in the Cont. group is described as 1 compared to the other groups. Significant differences are represented by different letters, *n* = 3. **d** Immunofluorescence images for MHC of p*MYOD1*-PEFs. The arrows marked a sarcomere-like structure. Scale bar = 100 μm

**Fig. 5:**
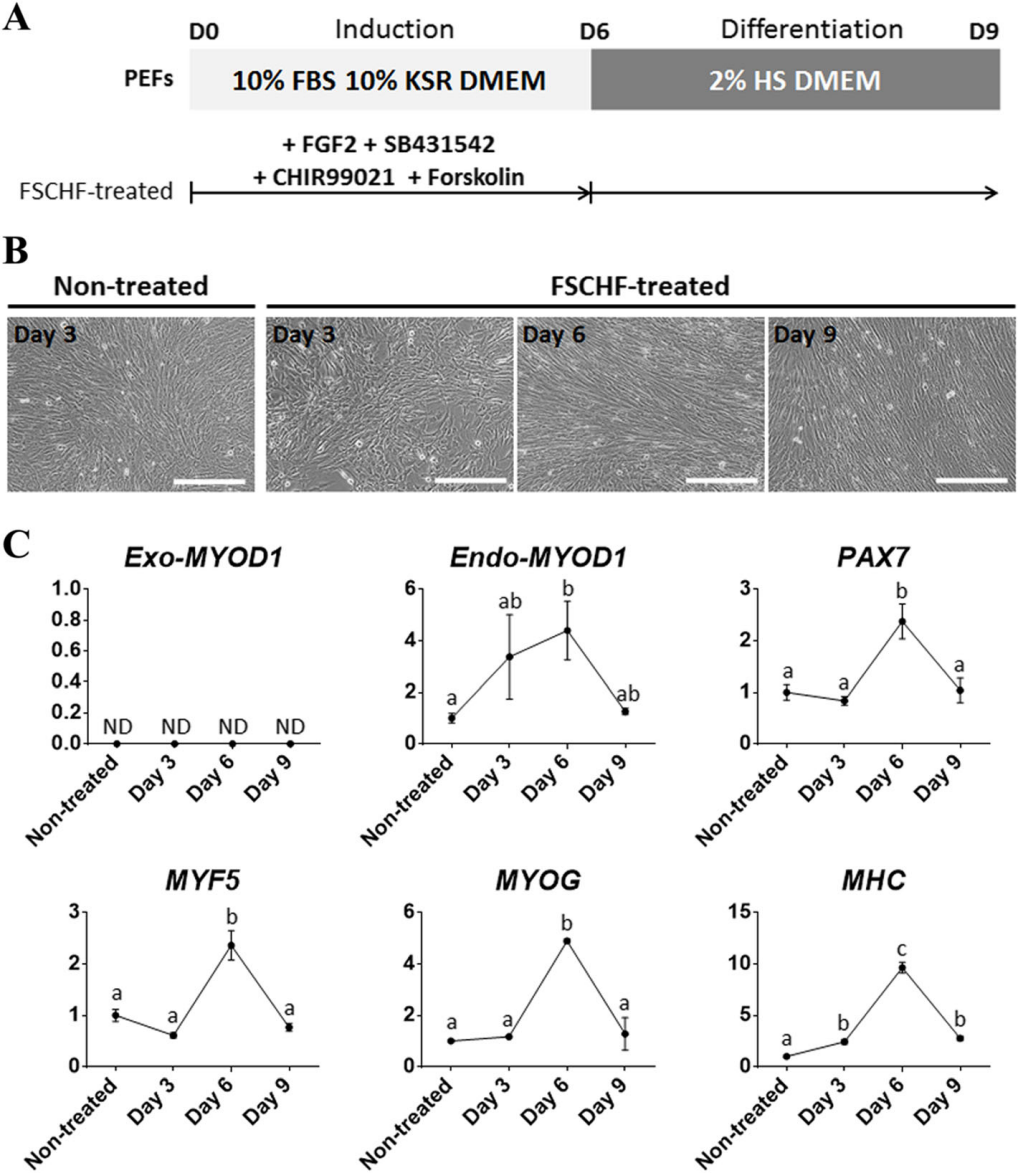
Treatment of signaling molecules without *MYOD1* overexpression in PEFs. **a** Experimental design. PEFs were cultured in the medium containing the FSCHF cocktail and the medium was replaced with a medium containing 2% horse serum without signaling molecules on day 6. The non-treated group contained PEFs which was cultured in basal media. **b** Cell images under respective culture conditions. Scale bar = 200 μm. **c** qPCR results to confirm the expression patterns of muscle-associated genes (*Exo-MYOD1, Endo-MYOD1, PAX7, MYF5, MYOG, *and* MHC*) in PEFs. The amount of gene expression in the non-treated group is described as 1 compared to the other groups. Significant differences are represented by different letters, *n* = 3

Based on the above observations, we established a myogenic transdifferentiation protocol in which the FSCHF medium for induction into a myogenic lineage was replaced with a low-serum medium for the initiation of differentiation on day 6 (Fig. 4a). The replaced culture condition was classified into two groups distinguished by the addition of DOX (+DOX and -DOX) to assess whether consistent activation of the myogenic program could enhance transdifferentiation. Notably, multinucleated myotubes via fusion of myoblasts were observed on day 8 in both groups (Fig. 4b). According to the qPCR analyses performed with a sample from day 9, transcripts of the *Exo-MYOD1, MYF5, MYOG*, and *Myosin heavy chain (MHC)* genes were upregulated in the +/− DOX groups (Fig. 4c). Unlike that of the other genes, gene expression of *Endo-MYOD1* was significantly decreased in the +/− DOX group. Across all of the genes, especially *MYF5* and *MHC*, the +DOX group showed significantly higher expression levels than the -DOX group, demonstrating that continuous *MYOD1* overexpression during the differentiation step facilitates myogenic transdifferentiation. The expression of MHC, a marker of late differentiation in myogenesis, was detected in the day 9 sample by immunofluorescence analysis (Fig. 4d). Interestingly, a sarcomere-like structure with a striated pattern was observed, as previously reported [35], indicating that the mature myotube could be assembled. Unless the *MYOD1* was ectopically expressed, the FSCHF cocktail was insufficient to trigger the activation of myogenesis-related genes in PEFs, indicating that the *MYOD1* plays a crucial role in determining the myogenic cell fate during transdifferentiation of the pig fibroblasts (Fig. 5). Therefore, the established protocol using ectopic *MYOD1* expression and signaling molecules associated with myogenesis, such as FGF2, a TGF-β inhibitor, a WNT activator, and a cAMP activator, enabled fibroblasts to be reprogrammed into skeletal muscle.

## Discussion

### Pig MYOD1 has an evolutionarily conserved bHLH domain that regulates myogenesis

Previously, numerous attempts have been made to reprogram non-muscle cells into skeletal muscle by modulating the expression of the transcription factor Myod1, which plays an important role in myogenesis [10, 11]. 5-azacytidine (5-aza), a DNA methyltransferase inhibitor, induces demethylation of the *Myod1* locus in differentiated somatic cells and increases *Myod1* transcripts, leading to transdifferentiation into the myogenic lineage [9, 36]. However, 5-aza does not target site-specific demethylation; it induces genome-wide demethylation because 5-aza is incorporated into genomic DNA as a competitive analog of cytosine and then disintegrates DNA methyltransferase by trapping [37]. In fact, when fibroblasts were treated with 5-aza, adipocytes and chondrocytes were also observed [38]. Additionally, it has been substantiated that 5-aza exhibits cytotoxicity by causing DNA double-strand breaks and apoptosis [39]. Thus, myogenic transdifferentiation using 5-aza is inappropriate in terms of safety and efficiency. As an alternative strategy, forced *Myod1/MYOD1* expression without induction by 5-aza has been used to activate the myogenic program in human and mouse fibroblasts [27, 34, 40]. In pig, myotubes were differentiated from induced pluripotent stem cells (iPSCs) via a combination of a 5-aza treatment and ectopically expressed *MYOD1* [17]. Although contractile porcine myotubes with sarcomeres were obtained from iPSCs within 11 days, the entire process would be more time-consuming because of the iPSC derivation period. Here, we established a direct transdifferentiation protocol with pig fibroblasts for the generation of skeletal muscle using porcine *MYOD1* overexpression and myogenesis-associated signaling molecules, bypassing the induction of the pluripotent state.

Comparative analysis of the MYOD1 amino acid sequence showed that the bHLH domain in MYOD1 was thoroughly conserved across various species. A previous study suggested that conserved sites are less permissive to evolutionary mutation due to their functional or structural importance [41]. This finding indicates that the bHLH domain is an essential part of the MYOD1 protein, the function of which is associated with myogenesis, as in other species [34, 42]. The basic region in the bHLH domain of Myod1 recognizes and binds the E-box, which is a conserved DNA sequence that is frequently distributed throughout the genome rather than specifically located in the regulatory region of myogenic genes [42]. The myogenic specificity of Myod1 is derived from Ala^114^- Thr^115^ in the basic region [29, 43]. In fact, it has been verified that Myod1 binds to both canonical and noncanonical E-boxes [10]. These myogenic codes direct Myod1 to bind to noncanonical E-boxes of *Myog*, one of the Myod1 target loci, and to interact with Pbx/Meis cofactors associated with myogenic genes [30]. In particular, Ala^114^ leads to an appropriate conformational change allowing myogenic activity by mediating the contact of Arg^111^ and guanine in DNA [31]. In addition to the basic region, the HLH motif of Myod1 is also required for myogenesis because the HLH motif dimerizes with other bHLH proteins, whose basic domains are involved in E-box binding [42]. The other domains are also highly conserved and have been known to be functional Myod1 domains [11, 44]. For example, the acidic domain acts as a transcriptional activation domain (TAD) through additional DNA binding near E boxes, and both the H/C and helix3 domains are involved in chromatin remodeling to allow active transcription of sequential myogenic genes. Accordingly, pig MYOD1 is capable of muscle-specific gene expression through conserved domains, especially the bHLH motif, preventing differentiation into non-muscle cell fate.

### Myogenic transdifferentiation is enhanced by activation of the FGF, WNT, and cAMP signaling pathways and inhibition of the TGF-β signaling pathway

Doxycycline (DOX)-inducible p*MYOD1* overexpression vectors were produced with the conserved pig MYOD1 sequence, as described above. Because differentiation is blocked when proliferation is enhanced by mitogen in serum, a low concentration of horse serum has been widely employed for myogenic differentiation in humans and mice [45–47]. However, transdifferentiated muscle cells were not observed when the aforementioned conventional culture conditions were applied. This result was consistent with the qPCR data that showed mild upregulation of endogenous muscle genes (Fig. 2c). It was surmised that myogenic reprogramming in pigs requires more supportive culture conditions than in mice and humans. For these reasons, a mixture of small molecules, such as FGF2, a TGF-β inhibitor (SB431542), a WNT activator (CHIR99021), and a cAMP activator (forskolin), was selected to facilitate the conversion of cell fate into the myogenic lineage, as applied in previous research [8]. While activation of the FGF, WNT, and cAMP signaling pathways is required for specification into the myogenic lineage and proliferation of committed myoblasts, inhibition of the TGF-β signaling pathway is involved in myotube formation [3, 13, 15]. These signaling molecules, except forskolin, are secreted during myogenesis *in vivo*, thus recapitulating the endogenous signaling pathway for muscle formation and regeneration. Forskolin has been reported to be involved in skeletal muscle differentiation from human iPSCs and to improve satellite cell expansion in mice [16]. Supporting the function of forskolin, our preliminary study showed that the removal of forskolin failed myogenic transdifferentiation (data not shown).

With the treatment of these four signaling molecules, endogenous myogenic genes were highly triggered by exogenous *MYOD1* (*Exo-MYOD1*) (Fig. 3c). Consistent with these observations, it has been demonstrated that ectopically expressed *MYOD1* upregulates endogenous *MYOD1* (*Endo-MYOD1*), *PAX7, MYF5*, and *MYOG*, which reinforces the notion that *MYOD1* is a key regulator in myogenesis. *Exo-MYOD1* increased *Endo-MYOD1* via an autoregulatory loop, as previously reported [26]. The temporal expression of myogenic genes has a hierarchy with stage-specific markers: *PAX7* in myogenic progenitor cells and *MYF5* and *MYOD1* in committed myogenic cells, followed by *MYOG* in the differentiation phase [3, 48]. In the present study, after day 9 from the Dox treatment, the expression of *Endo-MYOD1* and *MYF5* was increased while that of *PAX7* was undetected, which indicates the *PAX7* was involved in the early myogenic-transdifferentiation followed by the activation of the *MYOD1* and *MYF5* in the subsequent phase as like *in vivo* myogenesis. Chromatin immunoprecipitation reportedly showed that mesoderm or myoblast markers, including *Pax7/PAX7* and *Myog/MYOG,* were directly activated by *Exo-Myod1*, leading to an increase in *Myf5* [27, 49]. Because *MYF5* is upstream of *MYOD1*, it was reported that *MYF5* was not expressed in *MYOD1*-overexpressing human iPSCs [33]. However, when treated with signaling molecules, forced *Myod1* expression converted mouse fibroblasts into *Pax7*- and *Myf5*-positive myogenic progenitor cells [8]. Taken together, various cell types that belong to the myogenic lineage can be derived in mitogen-rich media. In the C2C12 myoblast cell line, mitogen-rich culture conditions stimulated myoblast growth without differentiation, whereas skeletal muscle was differentiated with the expression of differentiation-specific genes under low-mitogen culture conditions [50]. For the enrichment of terminally differentiated myotubes, the culture conditions were switched, and a medium containing 2% horse serum without signaling molecules on day 6 was used, in which endogenous muscle genes peaked by *Exo-MYOD1*.

As shown in the applied protocol presented in Fig. 4a, multinucleated and elongated myotubes were observed. These myotubes were formed through the fusion of mononucleated myoblasts exiting from the cell cycle, followed by a reorganization of the cytoskeleton [3]. Based on the higher expression of muscle genes in the +DOX group, continuous *MYOD1* overexpression seems to enhance myogenic conversion in a feed-forward mechanism [42]. Myotubes and myofiber express myosin heavy chain (MHC) [6], which is a downstream gene of Myod1 [26]. MHC provides contractility to eukaryotic cells through filament assembly in the form of striated sarcomeres, such as skeletal and cardiac muscle [51]. In fact, the expression of *MHC* was increased at the RNA level and a sarcomere-like structure was detected by immunostaining for MHC proteins in accordance with previous research [35]. Altogether, our protocol enables the transdifferentiated muscle to undergo terminal differentiation and maturation into skeletal muscle.

## Conclusions

In summary, fully differentiated somatic cells of pigs can be reprogrammed into mature skeletal muscle by the pig *MYOD1* gene. Modulation of the FGF, TGF-β, WNT, and cAMP signaling pathways is required for the cell fate conversion into the myogenic lineage. The transdifferentiated muscle expressed skeletal muscle markers and had the structure of a striated sarcomere, implying that these matured myotubes possess a contractile capacity. Given the role of pig as significant livestock for supplying meat, the myogenic reprogramming of pig cells can be applied to increase agricultural yield and produce cultured meat. Additionally, pigs provide biomedical applications in preclinical studies for human disease because of their anatomical and physiological similarities with humans [52, 53]. For example, transdifferentiated pig muscle cells can offer a cell source for skeletal muscle disease modeling and drug screening in regenerative medicine. Finally, this study provides fundamental knowledge for the developmental biology in revealing the genetic network and signaling pathways underlying myogenesis.

## Data Availability

Not applicable.

## Abbreviations

a.a: Amino acid
5-aza: 5-Azacytidine
bHLH: Basic-helix1-loop-helix2
Cont.: Control
DOX: Doxycycline
Endo-MYOD1: Endogenous MYOD1
ESCs: Embryonic stem cells
Exo-MYOD1: Exogenous MYOD1
FBS: Fetal bovine serum
FGF2: Fibroblast growth factor 2
FSCHF: FGF2, SB431542, CHIR99021, and forskolin
gDNA: Genomic DNA
H/C: Histidine and cysteine-rich
ICC: Immunocytochemistry
iPSCs: Induced pluripotent stem cells
KSR: Knockout serum replacement
MHC: Myosin heavy chain
pACTB: Porcine beta-actin
PEFs: Pig embryonic fibroblasts
pMYOD1: Pig MYOD1
pMYOD1-PEFs: PEFs infected with a lentivirus carrying the p*MYOD1* gene
PSCs: Pluripotent stem cells
qPCR: Quantitative real-time polymerase chain reaction
TAD: Transcriptional activation domain
